# Emerging mechanomedicines informed by mechanotransduction along the integrin–cytoskeleton–nucleus axis

**DOI:** 10.1063/5.0255473

**Published:** 2025-06-10

**Authors:** Yuka Yokoyama, Nya Domkam, Hannaneh Kabir, Abdullah Mansour, Shingo Tsukamoto, Ghafar Yerima, Taiji Adachi, Mohammad R. K. Mofrad

**Affiliations:** 1 Molecular Cell Biomechanics Laboratory, Departments of Bioengineering and Mechanical Engineering, University of California, Berkeley, California 94720, USA; 2 Department of Micro Engineering, Graduate School of Engineering, Kyoto University, 53 Shogoin-Kawahara-cho, Sakyo-ku, Kyoto 606-8507, Japan; 3 Department of Biosystems Science, Institute for Life and Medical Sciences, Kyoto University, 53 Shogoin-Kawahara-cho, Sakyo-ku, Kyoto 606-8507, Japan; 4 Molecular Biophysics and Integrative Bioimaging Division, Lawrence Berkeley National Lab, Berkeley, California 94720, USA

## Abstract

Mechanical forces are fundamental to the formation of normal biological tissues and the maintenance of physiological health. These forces are transmitted from the extracellular environment to the cell interior through cell–cell and cell–ECM interactions, the cytoskeleton, the LINC complex, the nuclear pore complex, and chromatin, ultimately regulating gene expression via transcription factors. This process, known as mechanotransduction, enables cells to convert mechanical signals into biochemical responses. Due to its critical role in various cellular functions and its influence on disease progression, mechanotransduction emerges as a potential therapeutic target for a range of conditions, including cancer and cardiovascular diseases, by integrating it with biochemistry, molecular biology, and genetics. Mechanomedicine, a burgeoning field, seeks to harness insights from mechanobiology to develop innovative diagnostic and therapeutic strategies. By targeting the molecular and cellular mechanisms underlying mechanotransduction, mechanomedicine aims to create more effective and precise treatments. Despite the potential, current clinical practices largely depend on conventional therapies like chemotherapy, underscoring the challenges of manipulating mechanotransducive pathways within living organisms. This review bridges fundamental mechanotransduction mechanisms with emerging therapeutic approaches, highlighting how mechanomedicine can revolutionize clinical practice. It explores the latest advancements in targeting mechanotransducive elements, discusses the therapeutic efficacy demonstrated in preclinical and clinical studies, and identifies future directions for integrating mechanobiological principles into medical treatments. By connecting basic mechanobiology with clinical applications, mechanomedicine holds the promise of offering targeted and reliable treatment options, ultimately transforming the landscape of disease management and patient care.

NOMENCLATURE

ACP: Actin cross-linking protein
AJ: Adherens junction
ATP: Adenosine triphosphate
ECM: Extracellular matrix
FA: Focal adhesion
G-actin: Globular actin
GAG: Glycosaminoglycan
GTPase: Guanosine triphosphatase
HGPS: Hutchinson–Gilford progeria syndrome
HP1: Heterochromatin protein 1
INM: Inner nuclear membrane
IPF: Idiopathic pulmonary fibrosis
JAM: Junctional adhesion molecule
KASH: Klarsicht, ANC-1, and Syne homology
LATS: Large tumor suppressor kinase
LINC: Linker of nucleus to cytoskeleton
LRMP: Lymphoid-restricted membrane protein
NE: Nuclear envelope
NPC: Nuclear pore complex
NTR: Nuclear transport receptor
ONM: Outer nuclear membrane
PG: Proteoglycans
PH: Pulmonary hypertension
Rho: RAS homolog
ROCK: Rho-associated coiled-coil Kinase
RV: Right ventricle
RVSP: Right ventricular systolic pressure
SUN: SAD1/UNC84
TAZ: Transcriptional co-activator with PDZ-binding motif
TEAD: TEA/ATTS domain
TGF-β: Transforming growth factor-beta
TJ: Tight junction
TRC: Tumor-repopulating cell
VGLL4: Vgl-like-4
YAP: Yes-associated protein

## I. INTRODUCTION

Mechanical forces are integral to both the development of biological tissues and the maintenance of healthy physiological functions. For example, mechanical strain in the aortic valve drives valvular interstitial cells to differentiate into calcific phenotypes, contributing to calcific aortic valve disease.^1–3^ Similarly, in the abdominal epidermis of pregnant mice, mechanical stretching induces vascularization and the formation of epidermal proliferative clusters, enabling skin expansion.^4,5^ Bone tissue also exemplifies this principle: forces acting on bone are sensed by osteocytes, which guide osteoblasts and osteoclasts to regulate bone structure and strength based on mechanical inputs.^6–8^ During development, insufficient mechanical loading on bones can lead to abnormal shapes and the premature fusion of articular bones.^9–11^ At a more fundamental level, processes such as cell migration and proliferation are orchestrated by cellular forces and their interactions with the environment.

**FIG. 1.**
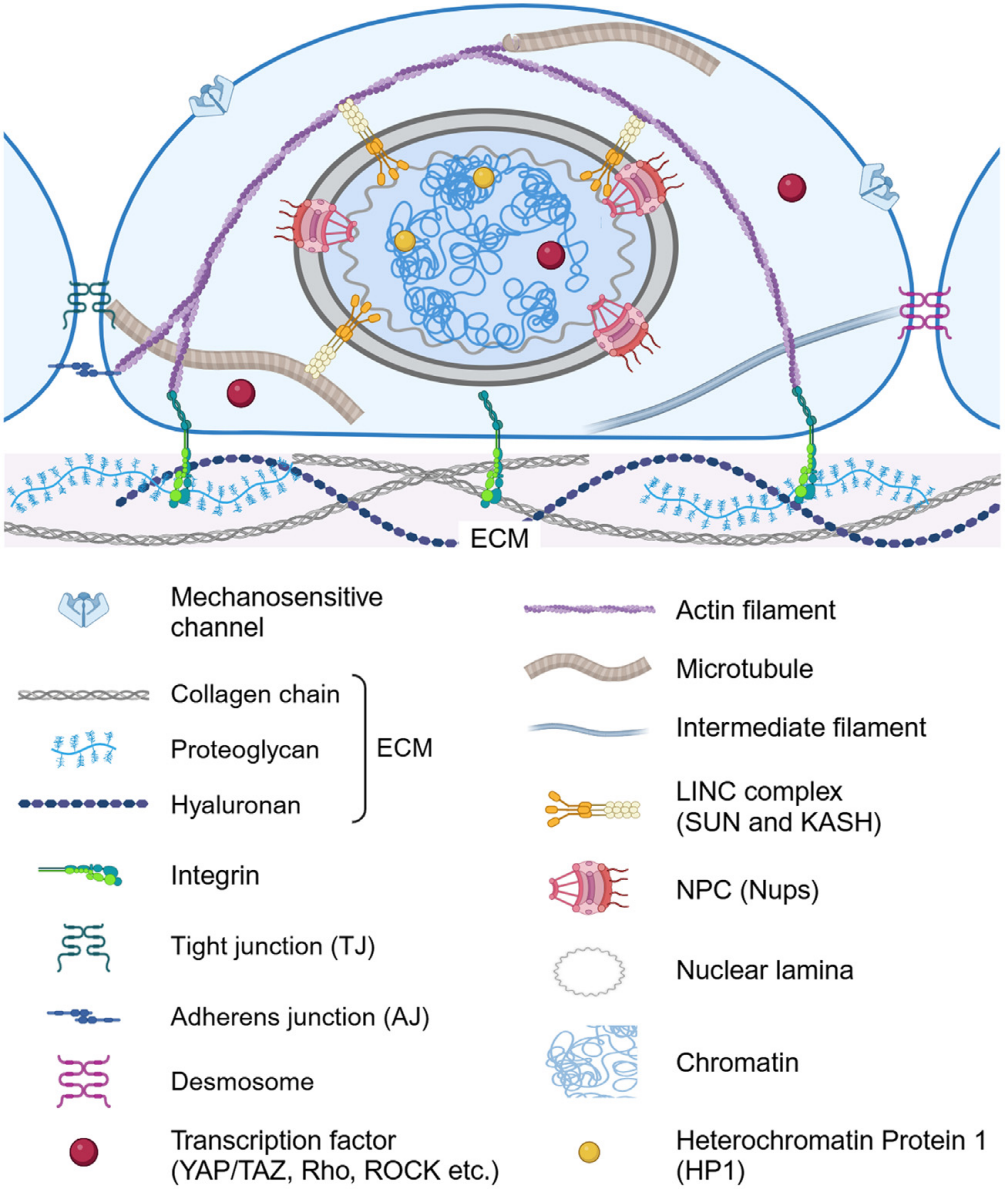
Key structural components and pathways involved in cellular mechanotransduction in a typical epithelial cell. Mechanical signals from the extracellular environment are relayed through mechanosensitive ion channels, cell–cell and cell–ECM adhesions, and the cytoskeleton. These forces then propagate through the LINC complex to influence the nuclear envelope, NPC, nuclear lamina, and chromatin. By translating physical cues into biochemical signals, these interconnected modules collectively regulate gene expression and underpin the emerging field of mechanomedicine.

These biological responses result from cellular mechanotransduction, the process by which mechanical signals are converted into biochemical cascades.^12–15^ Mechanotransduction operates through an interconnected network of intracellular components (Fig. 1). At the cell surface, mechanosensitive ion channels (e.g., PIEZO1, PIEZO2, TRPV2, and TRPV4) respond to membrane tension by triggering ion fluxes.^16,17^ Intercellular adhesions, particularly adherens junctions (AJs), enable force sensing between neighboring cells,^18,19^ while focal adhesions connect integrin receptors to the extracellular matrix (ECM).^20–24^

The cytoskeleton—comprising actin filaments, microtubules, intermediate filaments, and associated cross-linking proteins—functions as a mechanical force distributor throughout the cell.^25–28^ Through the LINC (Linker of Nucleoskeleton and Cytoskeleton) complex, which contains KASH and SUN proteins,^29–32^ these forces reach the nucleus.^33–35^ This mechanical signal propagation influences nuclear pore complex (NPC) function, affecting transcription factor translocation,^36,37^ and alters chromatin architecture, thereby modulating gene expression.^38,39^

Disruptions in mechanotransduction pathways frequently contribute to disease progression. Beyond calcific aortic valve disease, mechanical stimuli influence tumor invasiveness and cell motility.^40^ Musculoskeletal disorders, including osteoporosis, osteoarthritis, tendinopathy, muscle atrophy, and intervertebral disk degeneration, also arise from aberrant mechanical cues.^41^ These examples demonstrate how mechanical dysfunction exacerbates pathophysiological states.

In response to these insights, the field of *mechanomedicine* has emerged,^41–43^ seeking to leverage mechanobiological understanding for disease diagnosis and therapy. This new discipline aims to translate mechanotransduction principles into actionable strategies for diagnosing, monitoring, and treating diseases at their biomechanical roots. While current clinical practice relies heavily on chemotherapy, the sophisticated control offered by mechanotransduction mechanisms suggests opportunities for more targeted and effective therapeutic approaches.

Current therapeutic approaches face significant limitations in addressing mechanical dysfunction at cellular and subcellular levels. While several treatments have shown efficacy, most existing strategies fail to fully leverage recent advances in mechanobiology. The development of next-generation therapies requires sophisticated integration of mechanical principles into both drug design and delivery strategies (Table I). This integration must consider the complex interplay between mechanical forces and cellular responses across multiple scales.

**TABLE I.** Representative therapeutic approaches informed by mechanobiology. This table summarizes various treatment strategies that target mechanotransducive pathways or mechanosensitive molecules. By modulating components such as integrins, cytoskeletal filaments, the LINC complex, NPC, chromatin modifiers, or transcription factors, these interventions aim to correct aberrant mechanical signaling and restore healthy cellular function. These examples highlight the promise of mechanomedicine in providing more precise and effective disease management options.

Disease	Target	(Potential) Treatment	Efficacy
Cancer	Integrin αvβ3	Inhibition with small molecule antagonists to reduce tumor spread	Demonstrated in preclinical models^44^
	Abnormal actin–tropomyosin binding	Specific therapy by targeting actin–tropomyosin interactions	Demonstrated in cell culture experiments^45–48^
	SUN1	Removal of SUN1 destabilizes cell–cell junctions	Demonstrated in SUN1 knockout mouse models^49^
	SUN2	Targeting decrease in SUN2 mRNA in cancer	Demonstrated in CNS embryonal tumor cells^50,51^
	Sox2 gene	Silencing with HP1 as it relates to H3K9 methylation	Demonstrated in single-cell magnetic cytometry experiments^52^
	YAP/TAZ	Hyperactivation of YAP	Demonstrated in mouse models^53^
	YAP/TAZ–TEAD interaction	Disruption with ectopic expression of VGLL4 or IAG933	Demonstrated in rat and mouse models^54–56^
	ROCK1 and ROCK2 kinases	Inhibition with small molecule inhibitor AT13148	Demonstrated in cell culture and mouse models^57,58^
Fibrosis	Integrin αvβ6	Blockade with antibody	Demonstrated in mouse models^59^
	Fibrosis-suppressor gene	Inhibition of HP1 and G9a	Demonstrated in cell culture and mouse models^60^
	YAP/TAZ	Inhibition by verteporfin	Demonstrated in mouse models^61–63^
Alzheimer's disease	Integrin β1	Modulation with recombinant integrin β1 signal peptide	Demonstrated in cell culture and mouse models^64^
	Actin stabilization	Blocking cofilin phosphorylation by ROCK inhibitor, fasudil	Demonstrated in cell culture experiments^65^
	YAP	Activation by XMU-MP-1	Demonstrated in mouse models^66^
Heart failure	Integrin α5β1	Treatment with α5β1 signaling inhibitor ATN-161	Demonstrated in mouse models^67^
	Stable detyrosinated microtubules	Destabilization by high-dose colchicine	Demonstrated in several animal models^68–73^
	YAP/TAZ	Inhibition by statins	Demonstrated in mouse models^74^
Organ regeneration	SUN1	Recruitment of Drosha to the nuclear envelope	Demonstrated in mouse muscle transplant expirement^75^
	YAP	Forced activation of YAP	Demonstrated in mouse models^76–78^
Pulmonary hypertension	ECM stiffening	Reduction of ECM stiffness with LOX inhibitors	Demonstrated in mouse models^79^
	Rho/ROCK pathway	Inhibition with fasudil	Demonstrated in short-term clinical studies^80,81^
ALS	Actin barrier collapse	Treatment with humanized anti-RGMa monoclonal antibody	Demonstrated in mouse models^82^
ALS/FTD	Reduced expression of Nup50 and Gle1	Stabilization of NUPs by Sigma 1	Demonstrated in cellular and Drosophila models^83^
Diabetes	Integrin αvβ5	Blockade of αvβ5 integrin with antibodies	Demonstrated in mouse models^83,84^
Glomerular disease	YAP–TEAD interaction and activity	Suppression of YAP activity by verteporfin	Demonstrated in rat models^85^
HGPS	SUN2	Suppression through siRNA	Demonstrated in mouse model^86^
Metabolic disease	YAP	Increasing YAP abundance in the striated muscle	Demonstrated in mouse models^87^
Neurodegenerative diseases	ROCK	Inhibition with Y-27632 or fasudil	Demonstrated in rodent models^88^
Osteoarthritis	YAP	Suppression of YAP activity by siRNA, ROR2 silencing, or verteporfin	Demonstrated in mouse models^89–91^
Regenerative medicine	α-Actinin-4	Application for creation of strong and sufficiently deformable hydrogel materials	Concept proposed^92^
Virus infection	Nup	Defend from cleavage to maintain NPC filter functionality	Demonstrated in Huh-7 cells infected with the Zika virus^93,94^

The field urgently requires novel methodologies for studying cellular mechanics at subcellular resolution in living tissues. Current *in vivo* mechanical measurements provide only broad insights, necessitating more precise analytical tools. Future technological development must focus on quantitative assays that can measure mechanical properties such as cancer cell invasiveness relative to substrate stiffness and cytoskeletal organization. Advanced computational models capable of simulating complex mechanobiological systems will become crucial for drug discovery and optimization. Furthermore, standardized methods for measuring and monitoring mechanical interventions in clinical settings will ensure consistent treatment outcomes.

The future of mechanomedicine depends on the synthesis of insights from multiple fields. While mechanomedicine is a promising field, we must acknowledge that diseases are complex and can't be described by one methodology. There may be diseases that are more suited to be treated using chemotherapeutic methods, there may be some that are better suited for mechanomedicine, and some diseases may require a combination of different methodologies. The convergence of mechanics with biochemistry, molecular biology, and genetics has revealed intricate relationships between mechanical perturbations and cellular responses. This understanding enables the identification of force-sensitive residues and allosteric sites while suggesting novel applications for gene-editing tools in modifying cellular mechanical properties. The integration of these approaches will be essential for developing more effective therapeutic strategies.

This review examines mechanotransduction at both cellular and subcellular levels, highlighting how fundamental principles of mechanical signal conversion can inform the development of novel diagnostic tools and treatments in mechanomedicine.

## II. CELLULAR AND MOLECULAR COMPONENTS OF MECHANOTRANSDUCTION

### A. Cell–ECM and cell–cell interactions

Multicellular life depends on a supportive extracellular matrix (ECM) that provides a three-dimensional structural framework for the body. Cells reside within and are mechanically constrained by the ECM, and their behaviors are guided by both cell–ECM and cell–cell interactions. This section focuses on the adhesion complexes mediating these interactions and discusses how they influence mechanical responses, disease progression, and future therapeutic strategies.

#### 1. Cell–ECM interactions

The ECM consists of complex, tissue-specific macromolecular networks comprising collagen, elastin, hyaluronan, glycosaminoglycans (GAGs), proteoglycans (PGs), fibronectin, and laminin, as well as various matrix regulators and water (Fig. 1).^95,96^ Cells dynamically engage with the ECM through specialized cell–matrix adhesion complexes, including focal adhesions (FAs). Integrins—transmembrane receptors present in FAs—bind to ECM ligands and connect to the cytoskeleton via adaptor proteins such as talin and vinculin.^15,95,97,98^ FAs assemble in response to localized mechanical forces exerted by the ECM, and their stiffness may increase due to the reinforcement of FA–actin filament connections mediated by talin.^21,24,99^ The intracellular forces generated by actin polymerization and myosin II contractility are transmitted through integrins at FAs, ultimately guiding cell shape changes, migration, ECM stiffness sensing, and ECM remodeling.^97,100^

Altered mechanosensitivity in cell–ECM interactions contributes to a range of pathologies, including fibrosis, thrombotic cardiovascular disorders, and cancer. In idiopathic pulmonary fibrosis (IPF), excessive ECM deposition and the overexpression of integrin αvβ6 activate transforming growth factor-beta (TGF-β), driving fibrotic progression.^101^ To rephrase, cells exhibiting unrestricted ECM formation and mechanically adhering to the ECM increases mechanical stress. If we can find methods to either control the production of ECM or decrease the amount of integrin αvβ6-mediated ECM interactions, we can manage fibrotic progression. Clinical trials have demonstrated integrins' potential for more traditional therapeutic applications; however, the resulting outcomes change the mechanics of cells. One trial demonstrated that bexotegrast (PLN-74809), which inhibits the binding of αvβ6 integrin to latent TGF-β, can slow fibrosis progression and improve lung function in IPF patients.^101^ Inhibition of TGF-β affects the production of ECM-related proteins thus decreasing cell stiffness. Integrin-mediated interactions are also implicated in thrombus formation via the GPIIb/IIIa (integrin β3 family) receptor on platelets and megakaryocytes.^102,103^ Inhibition of GPIIb/IIIa–ligand binding has thus emerged as a powerful antithrombotic strategy, resulting in the clinical approval of tirofiban, eptifibatide, and abciximab for acute coronary syndromes and thrombotic events.^44^ These examples underscore the therapeutic potential of targeting the mechanical functions of cell–ECM adhesion complexes within the framework of mechanomedicine.

#### 2. Cell–cell interactions

Cell–cell junctions are specialized membrane domains that couple neighboring cells.^18,104^ These junctions are continuously formed and remodeled during tissue development. The primary junctional complexes are tight junctions (TJs), adherens junctions (AJs), and desmosomes (Fig. 1).^104–106^ TJs occur in various cell types, including epithelial, vascular endothelial, Schwann, and Sertoli cells.^104–107^ They are composed of integral membrane proteins, such as junctional adhesion molecules (JAMs), occludin, claudins, tricellulin, marvelD3, and cytoplasmic plaque proteins like ZO-1, ZO-2, ZO-3, and cingulin, which link membrane proteins to the cytoskeleton.^106^ TJs form selective barriers regulating ion, solute, and water permeability, thus maintaining distinct apical and basolateral domains.^108^

AJs rely on nectins and cadherins as core components.^104–106^ Cadherins mediate calcium-dependent adhesion, while nectins are calcium-independent and contain Ig-like loops with a PDZ-binding motif. AJs are crucial for the initial cell–cell contacts essential in embryogenesis and the long-term maintenance of tissue architecture. Desmosomes, another class of intercellular junctions composed of desmosomal cadherins, armadillo proteins, and plakins, anchor intermediate filaments at adhesion sites and distribute mechanical forces throughout the tissue.^104–106^

Disruption of TJs and AJs is implicated in various diseases, including cancers, chronic disorders, and neurological conditions.^109–111^ Consequently, research has turned to strategies that modulate these junctional proteins to either restore normal cellular function or inhibit disease progression. For instance, cadherin 11 is often overexpressed in invasive breast cancer cells; inhibiting cadherin 11 can reduce tumor cell migration and invasion *in vitro* and diminish tumorigenicity and growth in mouse models.^112^ Similarly, claudin-2, which modulates intestinal permeability, correlates with disease severity in inflammatory bowel disease. Suppressing claudin-2 tight junctions alleviates paracellular permeability increases and improves disease outcomes in experimental models.^113^ These findings underscore the potential of targeting cell–cell junctional components as part of the broader goals of mechanomedicine to combat human diseases.

### B. Cytoskeleton

The cytoskeleton is the principal structural framework of the cell, dictating its mechanical resilience and facilitating the transmission of forces from the extracellular matrix (ECM) to the nucleus.^15,32^ Connected to the ECM through focal adhesions (see Sec. II) and to the nuclear membrane via the LINC complex, the cytoskeleton ensures the efficient relay of mechanical signals across the cell. This section examines the structure and function of the cytoskeleton, its response to mechanical challenges, and its involvement in disease and potential therapeutic interventions.

The cytoskeleton comprises three main filamentous components—actin filaments, microtubules, and intermediate filaments—along with a variety of cross-linking proteins (Fig. 1). Actin filaments measure roughly 5–9 nm in diameter and form through the polymerization of globular actin (G-actin) monomers.^26,32^ These filaments resist tensile loads and form diverse higher-order assemblies with actin cross-linking proteins (ACPs) such as α-actinin and fascin.^32,92^ For instance, actin stress fibers connect to focal adhesions through α-actinin-mediated cross-linking, while the actin cortex—a thin, actomyosin-rich network beneath the plasma membrane—supports cellular shape and cortical tension.^26,27,114–116^

Microtubules are cylindrical structures composed of α- and β-tubulin subunits, with outer and inner diameters of about 25 and 17 nm, respectively.^32,117,118^ They are adept at resisting compressive and buckling forces due to their considerable flexural rigidity.^15,119,120^ Intermediate filaments, measuring 8–10 nm, possess mechanical properties that bridge the extremes of actin's tensile strength and microtubules' rigidity.^25,32,40^ Highly flexible and extensible, intermediate filaments can endure both tension and compression, adding resilience to the cellular structure.

Cytoskeletal elements, through their dynamic rearrangement, exhibit mechanical properties ranging from elastic gel-like behaviors to more viscous fluid-like responses.^15,28,118,121–125^ By modulating cross-linking levels and actin filament length distributions, the cytoskeleton fine-tunes cellular tension in response to mechanical cues.^115,116,126^ The cytoskeleton does not merely respond passively to external forces; it actively generates contractile forces essential for cellular activities such as migration and tissue remodeling. Myosin motor proteins convert adenosine triphosphate (ATP) into mechanical energy as they traverse actin filaments, inducing contractility within the cytoskeletal network.^127,128^ For migrating cells, heightened cortical tension at the cell's rear drives cortical flow and propulsive forces that move the cell forward, with actin cortex contraction also producing blebs that serve as leading-edge protrusions.^115,129^ Additionally, the maturity of actin stress fibers, influenced by substrate stiffness, regulates cell migration velocity.^130^ At the tissue scale, cytoskeletal dynamics underlie cell rearrangements and are integral to shaping tissue mechanical properties.^131–133^

Given the close relationship between cytoskeletal mechanics and cellular function, targeting these filaments offers potential avenues for diagnosis and therapy. In fact, the increase in cell deformability, which is associated with actin organization, directly correlates with the progression of a transformed phenotype from a benign cell to a malignant cancer cell.^134^ ECM stiffening enhances microtubule stability through glutamylation, thereby promoting breast cancer cell invasion.^40,135^ Moreover, distinct actin isoforms differentially modulate polymerization dynamics, migration, adhesion, and cytokinesis. Understanding these isoforms may enable the rational design of anti-cancer drugs that target aberrant actin signaling with reduced toxicity to normal cells.^45^

Cytoskeletal abnormalities also appear in other pathologies. For example, stable, detyrosinated microtubules are common in various heart failure conditions, and microtubule destabilization in animal models can yield cardioprotective effects—albeit with limited therapeutic indices due to on-target toxicity.^136,137^ Beyond pathology, cytoskeletal insights have implications in regenerative medicine. For instance, the load-dependent binding lifetime of α-actinin-4 to actin filaments (shorter at low loads and longer at intermediate loads) enables dynamic redistribution of cross-linkers within the cytoskeletal network.^92^ This mechanism contributes to the mechanical robustness and adaptability of actin networks and inspires the design of bioinspired hydrogel materials for tissue engineering and regenerative therapies.

Understanding the cytoskeleton's structure, mechanics, and regulatory mechanisms reveals numerous opportunities to modulate cell behavior, identify pathological states, and engineer new therapeutic strategies. By integrating these insights, mechanomedicine can harness the cytoskeleton's mechanical functions to develop advanced diagnostics and therapeutics for diverse diseases.

## III. NUCLEAR MECHANOTRANSDUCTION MODULES

### A. LINC complex

This section examines the Linker of Cytoskeleton and the Nucleus (LINC) complex, highlighting its structural components—particularly the SUN and KASH proteins—and its function as a mechanical force conduit between the cytoskeleton and the nucleoskeleton. We also discuss diseases associated with LINC complex dysfunction and explore its potential as a therapeutic target.

**FIG. 2.**
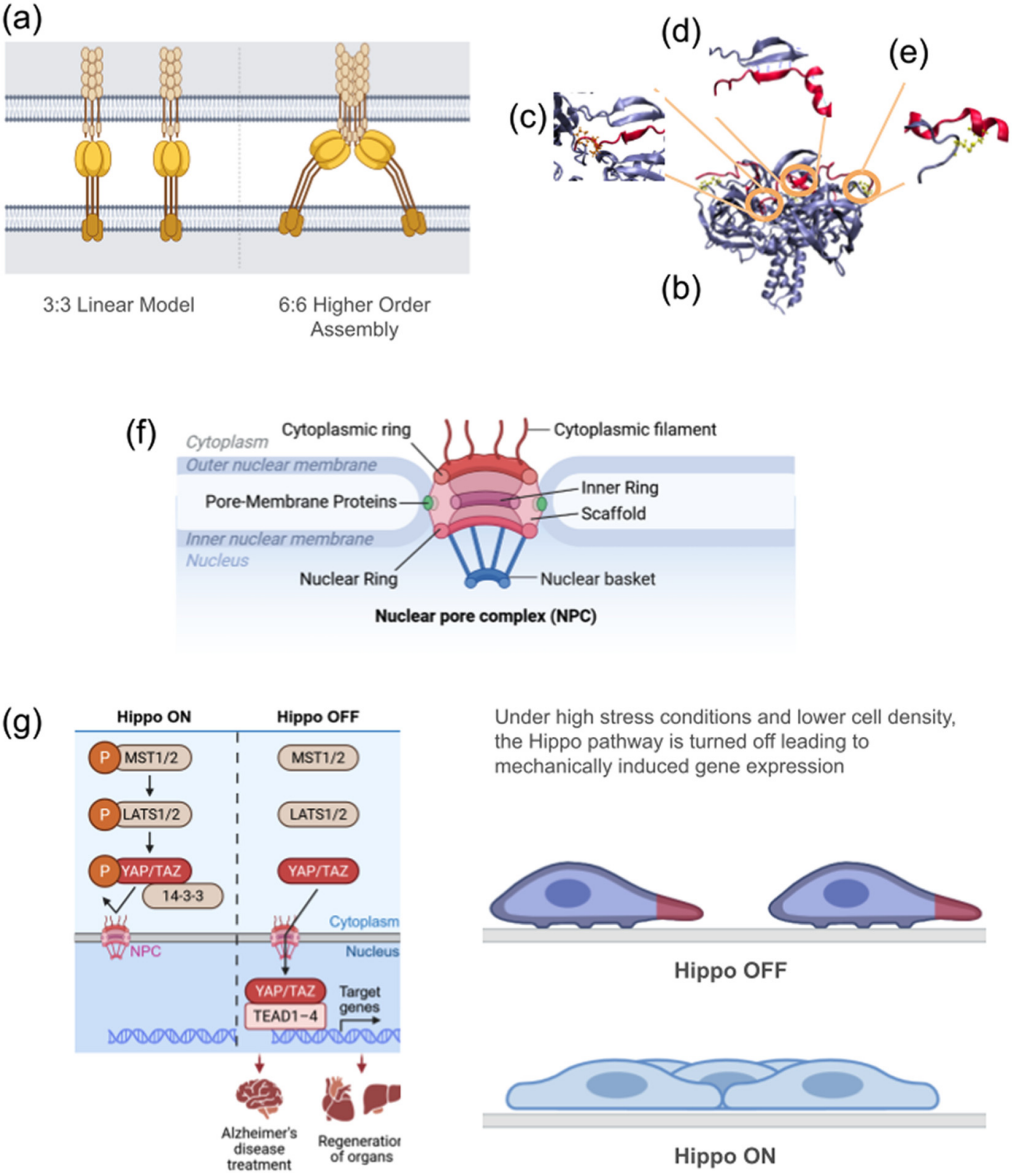
Architectural organization and force-transducing interactions within the LINC complex. (a) The LINC complex arranges itself in two forms: linear model and higher-order assembly. The linear model is a trimer and contains three SUN and three KASH protomers. It is sometimes referred to as the 3:3 model. The higher-order model consists of two 3:3 models coming together such that the KASH are interacting with each other. (b) There are three interactions that keep the LINC complex. The first interaction (c) is the PPPX motif of KASH interacting with a hydrophobic pocket of SUN. The next interaction (d) is hydrogen bonds that form between KASH and a section of SUN known as the KASH lid. The final interaction (e) is only seen in two different but most common KASH isoforms. This is a disulfide interaction between the SUN and KASH. (f) Structural organization of the nuclear pore complex (NPC). This cross-sectional schematic shows the NPC's characteristic eightfold symmetry, extending from the cytoplasmic filaments on the cytoplasm-facing side to the nuclear basket on the nucleoplasm-facing side. The central channel, situated between the cytoplasmic and nuclear rings, is lined with phenylalanine-glycine (FG)-repeat nucleoporins (FG-Nups). These FG-Nups form a selective permeability barrier that regulates nucleocytoplasmic transport and can be influenced by mechanical cues. By controlling the passage of transcription factors and other cargoes, the NPC serves as a critical mechanotransducive element, offering promising targets for mechanomedicine-based interventions. (g) Mechanosensitive regulation of gene expression via the Hippo-YAP/TAZ pathway. In the canonical Hippo pathway, the MST1/2 kinase complex phosphorylates and activates LATS1/2 kinases, which in turn phosphorylate YAP/TAZ. When phosphorylated, YAP/TAZ bind 14-3-3 proteins and remain cytoplasmic, preventing their nuclear entry. Under mechanically permissive conditions (e.g., high ECM stiffness or low cell density), YAP/TAZ avoid phosphorylation and translocate to the nucleus, where they associate with TEAD transcription factors to modulate gene expression. This mechanosensitive signaling axis is central to tissue homeostasis, regeneration, and disease. Harnessing YAP/TAZ activity offers potential therapeutic strategies in mechanomedicine, including treatments for neurodegenerative conditions such as Alzheimer's disease and promoting tissue regeneration in organs with limited intrinsic repair capacity. The orange “P” denotes phosphorylation events.

The LINC complex is a key molecular assembly bridging cytoskeletal filaments and the nuclear interior.^138,139^ It consists primarily of two protein families that span the inner and outer membranes of the nuclear envelope (NE). SUN (Sad1 and UNC84 homology) domain proteins reside at the inner nuclear membrane (INM), while KASH (Klarsicht, ANC-1, and Syne homology) domain proteins are anchored at the outer nuclear membrane (ONM).^140^ KASH domains are typically part of nesprins, large nuclear envelope spectrin repeat proteins that extend to cytoskeletal elements including actin filaments, microtubules, and intermediate filaments.^141,142^ Five SUN isoforms (SUN1–5) and six KASH proteins (nesprins 1–4,^143,144^ KASH5,^145^ and lymphoid-restricted membrane protein [LRMP]^146,147^) have been identified. These SUN and KASH proteins associate to form trimeric complexes at the NE.^141,148,149^ This arrangement is often referred to as the “3:3 linear model,” in which three SUN protomers interact with three KASH protomers [Figs. 2(a) and 2(b)].

Recent findings suggest that SUN–KASH complexes can also assemble into higher-order architectures. One proposed model is a 6:6 assembly, where two trimeric units form a larger complex [Fig. 2(a)].^150^ Subsequent work has identified an asymmetric 9:6 arrangement, featuring three SUN trimers each bound to two distinct KASH peptides.^29^ Molecular dynamics simulations indicate that these higher-order structures enhance nuclear envelope stability and that multiple configurations may coexist depending on cellular conditions and mechanical load.^31^

The LINC complex functions as a crucial mechanotransducer, conveying force through the nuclear envelope to influence an array of cellular processes. SUN and KASH domains interact in distinct ways: all KASH proteins share a conserved PPPX motif that fits into an SUN hydrophobic pocket [Fig. 2(c)], while hydrogen bonds link KASH domains to the “KASH lid” region of SUN [Fig. 2(d)]. In KASH1 and KASH2, an additional disulfide bond with SUN [Fig. 2(e)] further stabilizes their interaction. Disrupting this disulfide bond substantially reduces the LINC complex's mechanical load-bearing capacity.^31,151^ These SUN–KASH interactions act as “force gates,” controlling force transmission through the LINC complex. Variations in KASH domain proteins, which connect to different cytoskeletal filaments, allow for differential force conduction across cell types. Moreover, SUN/KASH complexes may form clusters or networks spanning the NE, enhancing nuclear mechanical resilience.^31^

As a central mediator of mechanotransduction, the LINC complex regulates nuclear positioning, shape, cellular proliferation, motility, and gene expression.^152–154^ Perturbations in LINC complex components are linked to conditions affecting fertility, vision, neurodegenerative disorders, muscular dystrophy, and laminopathies.^155–158^ Two illustrative examples are hearing loss and Hutchinson–Gilford progeria syndrome (HGPS). In certain forms of hearing loss, mutations in KASH4 prevent proper linkage to microtubules, impeding force transmission to the nucleus in cochlear outer hair cells.^159,160^ While there are currently no approved treatments targeting the LINC complex, potential therapies might focus on restoring LINC-cytoskeleton functions. Through protein engineering, we may affect nuclear positioning by connecting microtubules to the nucleus.

In contrast, HGPS involves excessive force transmission. Although caused by a lamin mutation, HGPS pathogenesis also relates to the LINC complex, as mutated lamin alters SUN2 distribution and consequently the arrangement of actin filaments attached to the nucleus.^161^ Reducing LINC complex expression can partially normalize nuclear shape and mitigate DNA damage in HGPS cells. Through reducing the amount of linkers between the nucleus and cytoskeleton, we can prevent stress-induced DNA damage. While wholly suppressing SUN2 may be problematic due to its ubiquity, more nuanced interventions that tweak SUN2 functionality or alter force transmission properties may offer safer therapeutic avenues.

As a structural linchpin of cellular mechanics with ties to diverse pathologies, the LINC complex holds tremendous promise for mechanomedicine. With continued advances in our understanding of its assembly, force transmission, and disease associations, the LINC complex is poised to become a focal point for developing novel therapeutic strategies targeting cellular mechanics and nuclear architecture.

### B. Nuclear pore complex

The nuclear pore complex (NPC) is a large multiprotein channel embedded in the nuclear envelope, serving as the principal gateway between the cytoplasm and nucleoplasm. It permits the free diffusion of small molecules while selectively regulating the transport of larger cargoes. This section reviews the structure of the NPC, the factors influencing its transport mechanisms, and the connections between NPC function, mechanotransduction, disease, and potential therapeutic strategies.

The NPC is composed of three main architectural regions, each built from distinct nucleoporin proteins (Nups) [Fig. 2(f)].^162^ On the cytoplasmic side, cytoplasmic filaments and the cytoplasmic ring project into the cytoplasm. Deeper within the nuclear envelope, the inner ring and channel nucleoporins span the transmembrane region of the pore. Many channels nucleoporins harbor phenylalanine-glycine (FG) repeats, earning them the shorthand “FG-Nups,” and these repeats form a selective permeability barrier that regulates nucleocytoplasmic transport. On the nucleoplasmic side, the nuclear basket and the nuclear ring extend toward the interior of the nucleus. Together, these components anchor the NPC to the nuclear envelope via pore membrane proteins.^162^

The NPC's overall architecture exhibits an eightfold rotational symmetry, a pattern that is consistently maintained from the cytoplasmic filaments to the nuclear basket.^163,164^ This symmetry reflects its robust structural design, ensuring that the NPC can efficiently coordinate cargo transport under varying physiological conditions. Understanding how the NPC's architecture and FG-Nup composition influence selective transport is essential for deciphering mechanosensitive pathways and identifying how mechanical cues may modulate transport efficiency.

The NPC's role extends beyond passive transport; it is also a key player in mechanotransduction, linking mechanical forces to changes in nucleocytoplasmic exchange. Mechanical deformation of the nuclear envelope can modulate NPC conformation, altering the permeability barrier and transport kinetics of FG-Nups. For example, forces transmitted via the LINC complex to the nuclear envelope can influence NPC gating and selectively regulate the translocation of mechanosensitive transcription factors, such as YAP and TAZ.^36,37^ Additionally, changes in cytoskeletal tension can reshape nuclear architecture, indirectly affecting NPC functionality and nucleocytoplasmic exchange rates.

Dysfunction in NPC components has been implicated in a range of diseases, including cancer, neurodegenerative disorders, and aging-related conditions. Mutations in Nups or disruptions in FG-Nup composition can compromise the selective barrier, leading to aberrant transport and altered nuclear signaling. For instance, mislocalization of FG-Nups has been observed in certain cancers, where disrupted nucleocytoplasmic transport contributes to unchecked cellular proliferation. Similarly, age-related NPC deterioration, characterized by FG-Nup depletion, has been linked to nuclear envelope leakiness and compromised genomic integrity.^165,166^

Treating the NPC like a specialized molecular filter can inspire novel therapeutic strategies for addressing pathology relating to expression. Small molecules designed to stabilize FG-Nup interactions or modulate NPC gating could restore transport selectivity in disease contexts. There have been studies that have looked at the effect of protease inhibitors on virus such as zika (Table I). The mechanism of action for viral genes using cell machinery is proteolysis of different nups. As a result, the NPC loses its ability to be selective. Likewise, we can potentially use this method to modulate NPC selectivity. Furthermore, understanding NPC mechanosensitivity provides opportunities to harness mechanical cues for precise control of the nucleocytoplasmic exchange, offering new avenues for drug delivery and therapeutic interventions.

### C. Chromatin

Mechanical forces transmitted from the extracellular matrix (ECM) through cell adhesion sites, the cytoskeleton, and ultimately via the LINC complex and the NPC eventually reach the nucleus and its chromatin, which houses the cell's genetic information.^33,35^ Structural changes in the nucleus and chromatin induced by external mechanical forces significantly affect gene transcription.^167,168^ Because nuclear and chromatin-level mechanical responses have a direct impact on genomic activities, the nucleus is often considered a major cellular mechanosensor.^169^ This section focuses on chromatin mechanics and examines how chromatin structure, mechanical signals, and epigenetic modifications relate to disease states and potential therapeutic strategies.

The nucleus is notably stiff compared to other organelles, with measured stiffness values ranging from approximately 0.1–10 kPa.^170–173^ Nuclear lamina and chromatin have traditionally been regarded as the key structural and mechanical supports of the nucleus.^34,174^ Their mechanical roles differ: chromatin primarily governs nuclear deformation responses under small strains (<30%), while lamin A/C dominates the response to large strains (>30%).^175^ For instance, a 10% cell–substrate stretch—akin to physiological conditions like vasoconstriction—produces about 1%–3% intranuclear strain in HeLa cells.^176^ While the roles of lamins in nuclear mechanics and disease are well established,^177–179^ this section emphasizes chromatin's influence. Moreover, chromatin-associated proteins have emerged as critical chromatin cross-linkers that enhance nuclear stiffness and mechanical stability.^180–183^ For example, heterochromatin protein 1 (HP1) mediates chromatin condensation and cross-linking, thereby increasing nuclear stiffness.^182,184^

**FIG. 3.**
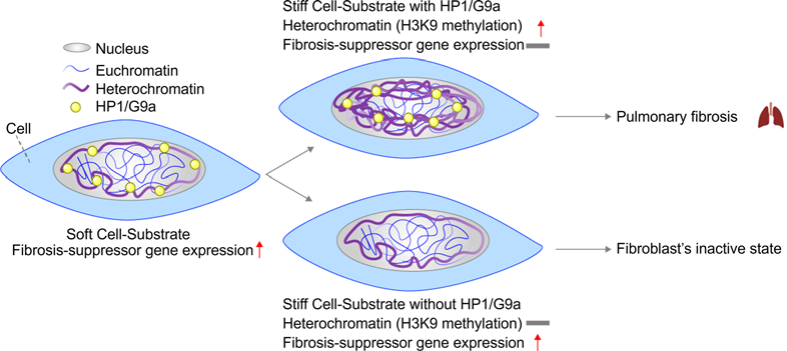
Mechanosensitive epigenetic regulation in pulmonary fibrosis.^60^ This schematic illustrates how lung fibroblasts cultured on a stiff substrate enhance H3K9 methylation, a heterochromatin marker associated with transcriptional silencing and fibroblast quiescence. Epigenetic mechanotransduction through factors such as heterochromatin protein 1 (HP1)α and the histone methyltransferase G9a translates mechanical cues from matrix stiffness into H3K9 methylation changes. This mechanosensitive process influences fibroblast activation and contributes to the pathogenesis of pulmonary fibrosis. By targeting these mechano-epigenetic pathways, mechanomedicine offers new therapeutic avenues for preventing or mitigating fibrotic diseases.

Mechanotransduction at the chromatin level affects cellular behavior and can contribute to various diseases. Changes in three-dimensional chromatin structure can disrupt enhancer–promoter contacts, resulting in dysregulated gene expression associated with cancer, autoimmune disorders, chronic kidney disease, and congenital limb malformations.^185–191^ Chromatin's epigenetic state can shift in response to mechanical cues, notably through histone modifications.^192^ For example, on stiff substrates where increased nuclear tension is expected, lung fibroblasts exhibit elevated levels of H3K9 methylation—a heterochromatin mark that represses gene expression.^60^ This mechanosensitive epigenetic change supports the fibroblast's quiescent state, and its absence can lead to pathological conditions such as pulmonary fibrosis, characterized by active fibroblasts and progressive scarring. Inhibiting HP1 or the histone methyltransferase G9a prevents stiffness-induced H3K9 methylation and restores the expression of fibrosis-suppressor genes (Fig. 3).^60^

In cancer, differentiated melanoma cells increase H3K9 methylation in response to stiff matrices, whereas tumor-repopulating cells (TRCs) do not. By maintaining low levels of H3K9 methylation, TRCs continue to express the Sox2 gene, preserving their self-renewal capacity.^52^ These findings highlight the importance of mechanosensitive heterochromatin states in disease progression. Notably, HP1's force-sensitive behavior suggests that targeting mechanosensitive chromatin-associated proteins could be a promising strategy for treating diseases influenced by mechanical cues. Recent molecular dynamics simulations have shown that under tensile force, the HP1–HP1 dimer interface becomes more stable due to a force-induced salt bridge formation, reinforcing the concept of HP1 as a mechanosensitive element.^38^ Although techniques like forced chromatin looping using artificial zinc-finger proteins have shown therapeutic potential in conditions such as sickle cell anemia,^193^ the broader field of mechano-genome therapy remains largely unexplored.

Both experimental and computational studies of chromatin mechanics will advance our understanding of epigenetic responses to mechanical forces, guiding the identification of new therapeutic targets such as HP1. Ultimately, these efforts may culminate in novel mechano-genome therapies that harness the interplay between force and the genome to treat a wide range of diseases.

## IV. TRANSCRIPTIONAL RESPONSE MODULES

Forces transmitted through cell–ECM and cell–cell adhesions, the cytoskeleton, the LINC complex, the NPC, and chromatin eventually influence gene expression. Such mechanotransducive pathways affect transcription factors that guide cell fate and function. This section reviews how transcriptional regulators—including YAP/TAZ, Rho, and ROCK—translate mechanical signals into gene expression changes, their connections to disease, and potential therapeutic applications.

### A. YAP/TAZ

YAP (Yes-associated protein, also known as YAP1) and its paralog TAZ (transcriptional co-activator with PDZ-binding motif) are key transcriptional co-activators controlling genes involved in proliferation, migration, cancer metastasis, survival, and stemness.^194–196^ YAP/TAZ activity is governed by the mechanosensitive Hippo signaling pathway [Fig. 2(g)].^197–200^ The pathway is initiated by MST1/2 kinases (MST1 and MST2, also known as STK4 and STK3), which activate LATS1/2 kinases (LATS1 and LATS2).^201^ Activated LATS1/2 phosphorylates YAP/TAZ, promoting their retention in the cytoplasm via binding to 14-3-3 proteins and preventing their nuclear entry. Conversely, when MST1/2 and LATS1/2 are inactive, unphosphorylated YAP/TAZ translocate into the nucleus, where they bind TEAD (TEA/ATTS domain) transcription factors (TEAD1–4) to drive gene expression.^201^

The Hippo pathway's responsiveness to mechanical cues makes it a crucial node of mechanotransduction. Under stiff ECM conditions or low cell density, LATS1/2 phosphorylation is downregulated, enabling YAP/TAZ nuclear localization and transcriptional activation.^201,202^ When cell density is high, leading to many adherens junctions, LATS1/2 phosphorylation increases and YAP/TAZ remains cytoplasmic.^203–206^ Additionally, as discussed earlier, nuclear membrane tension can modulate NPC conformation, facilitating the nuclear import of YAP/TAZ.^36^ Thus, mechanical signals from the extracellular environment and cell shape changes translate directly into transcriptional regulation by YAP/TAZ.

YAP/TAZ's central role in controlling essential cellular activities makes them attractive therapeutic targets.^207^ Current therapeutic approaches targeting the YAP/TAZ–TEAD interaction have evolved through several mechanistic strategies. Direct inhibition through VGLL4 mimetics has shown promise in blocking the YAP/TAZ–TEAD interface.^54,208^ Small molecule intervention, particularly with compounds such as IAG933, has demonstrated effectiveness in disrupting transcriptional complex formation.^55,56^ Upstream pathway modulation offers additional control points for therapeutic intervention. The integration of these approaches with existing cancer treatments has emerged as a promising direction for comprehensive cancer therapy.

YAP activation has demonstrated significant therapeutic potential across multiple regenerative contexts. In neurodegeneration, controlled YAP activation improves cognitive outcomes in Alzheimer's models through mechanisms involving neural plasticity and survival.^66^ The regenerative capacity of YAP extends to adult cardiac tissue repair, aged liver regeneration, and intestinal epithelium renewal.^76,194^ Each application requires precise control of YAP activity to maximize therapeutic benefit while minimizing potential adverse effects.

Next-generation therapeutics development focuses on several key areas. Tissue-specific delivery systems incorporate nanoparticle-based targeting, cell-type-specific expression systems, and mechanical environment-responsive activation mechanisms. Temporal control mechanisms utilize inducible expression systems and reversible inhibitors to achieve precise therapeutic windows. Safety enhancement strategies include restricted activation domains and feedback-controlled systems that respond to tissue mechanical properties.

The successful implementation of YAP/TAZ-targeted therapies requires sophisticated monitoring and optimization approaches. Biomarker development for patient stratification enables more precise therapeutic targeting. Real-time monitoring of pathway activation allows for dynamic treatment adjustment. The adaptation of treatment protocols based on mechanical tissue properties ensures optimal therapeutic response. Furthermore, careful consideration of dosing and timing helps prevent adverse effects while maintaining therapeutic efficacy.

### B. Rho GTPase and ROCK pathways

Rho (RAS homolog) GTPases belong to the Ras-like small G-protein family, encompassing more than 20 proteins, including Rho, Rac, and Cdc42.^106,209,210^ These proteins function through a molecular switch mechanism, transitioning between GTP-bound active and GDP-bound inactive states.^209,210^ Their activation leads to the subsequent activation of Rho-associated coiled-coil kinases (ROCKs), which exist in two isoforms: ROCK1 (p160ROCK/ROKβ) and ROCK2 (Rho-kinase/ROKα).^80,211^

The pathway exhibits mechanosensitive regulation through cell–cell adhesions, particularly in the case of Rac and Cdc42, which respond to E-cadherin-mediated contacts.^212–214^ This mechanical sensitivity manifests in distinct cellular responses: Rac governs actin polymerization and integrin adhesion complex formation,^215^ while ROCK proteins control actin–myosin contractility and cytoskeletal organization.^216^ These mechanisms collectively regulate cellular behaviors including motility, division, adhesion, polarity, and migration.^80,211,217–219^

Rho GTPase dysregulation plays a significant role in cancer progression, making these proteins attractive therapeutic targets. Different cancers show characteristic patterns of Rho GTPase expression: breast, gastric, and testicular cancers exhibit elevated Rac1 levels, while lung, breast, and colon cancers show increased RhoA expression.^209,220,221^

Several therapeutic strategies have emerged targeting these pathways. The Rac-specific inhibitor NSC23766 demonstrates anti-cancer effects through multiple mechanisms: suppressing invasion and anchorage-independent growth in prostate cancer PC-3 cells while inducing cell cycle arrest and apoptosis in breast cancer cells.^220,222^ Complementarily, the Cdc42-selective inhibitor AZA197 shows promise in colon cancer treatment by restructuring the actin cytoskeleton and inhibiting cell proliferation, migration, and invasion.^223^

ROCK inhibition has emerged as a promising therapeutic strategy for cardiovascular disorders.^211^ Preclinical studies in pulmonary hypertension (PH) using the ROCK inhibitor fasudil have shown significant therapeutic benefits, including reduced right ventricular systolic pressure (RVSP), decreased right ventricular hypertrophy, and improved arterial histopathology.^81^ These findings have translated successfully to clinical applications, with short-term studies in PH patients confirming fasudil's therapeutic efficacy.^80^

Recent developments have expanded the therapeutic potential of ROCK inhibition. The novel inhibitor AT13148 demonstrates efficacy in pancreatic cancer by targeting collagen invasion through modulation of cell motility and contractility.^57,58^ Furthermore, combination therapy approaches show particular promise.^224^ The integration of fasudil with tranilast and temozolomide enhances therapeutic outcomes in glioblastoma models through the promotion of neuronal reprogramming.^58^ These findings suggest that ROCK inhibition may serve as both a primary therapeutic strategy and a means to enhance the efficacy of existing treatments across multiple disease contexts.

## V. CLINICAL IMPLEMENTATION AND FUTURE DIRECTIONS IN MECHANOMEDICINE

Mechanotransduction pathways represent a frontier in therapeutic intervention. Throughout this review, we have examined multiple cellular components that offer promising therapeutic targets. Cell–ECM and cell–cell adhesion molecules, particularly integrins and cadherins, have emerged as crucial intervention points in cancer metastasis treatment. The disruption of these adhesion molecules effectively halts mechanical signal propagation and suppresses disease-promoting protein expression. In the cytoskeletal domain, approaches such as ROCK inhibition and microtubule destabilization demonstrate therapeutic potential, despite persistent challenges with off-target effects. The nuclear mechanotransduction machinery, particularly the LINC complex and NPC, presents novel opportunities for treating conditions such as laminopathies and muscular dystrophies, where mechanical force transmission plays a central pathological role.

Advanced mechanomedicine approaches must address several key challenges. Targeting strategies require enhanced specificity for mechanical intervention to minimize systemic effects (Table II). Cytoskeletal manipulation techniques need refinement to reduce off-target effects while maintaining therapeutic efficacy. Nuclear mechanotherapy demands precise control of force transmission through molecular engineering approaches. Additionally, delivery systems for mechanical modulators must be optimized for tissue-specific targeting and controlled release.

**TABLE II.** FDA-approved therapeutic approaches informed by mechanobiology. The approved targeting landscape includes integrins and kinases that affect signaling that influences the mechanical properties of cells.

Disease	Target	(Potential) Treatment
Fibrosis	ROCK2	Inhibition with Belumosudil^225^
Acute coronary syndrome and thrombotic cardiovascular events	Integrin αIIbβ3	Prevention of platelet aggregation by inhibiting binding to fibrinogen by antagonist, RGD mimetic, or Pan-β3 antagonist^44^
Dry eye disease	Integrin αLβ2	Treatment with αLβ2 (LFA-1) antagonist^44^
Glaucoma	ROCK2	Inhibition with Ripasudil^226,227^
Multiple sclerosis and Crohn's disease	Integrin α4β7 and α4β1	Inhibition of ligand binding by Pan-α4 antagonist^44^
Plaque psoriasis	Lymphocyte-specific integrin αLβ2	Prevention of lymphocyte activation and migration by αL antagonist^44^
Ulcerative colitis and Crohn's disease	Integrin α4β7	Inhibition of binding to MADCAM1 by antagonist^44^

The translation of mechanomedicine insights into clinical practice requires the systematic development of several components. Standardized protocols for mechanical assessment must be established across different tissue types and disease states. Integration with existing treatment modalities demands careful consideration of timing and dosing strategies. The development of reliable mechanical biomarkers will enable better patient monitoring and treatment optimization. Clinical guidelines for mechanical intervention need to be established through rigorous trials and validation studies.

This review has examined the complex landscape of cellular mechanotransduction and its therapeutic implications. From the extracellular matrix to nuclear mechanics, each cellular component presents unique opportunities for therapeutic intervention. The emergence of mechanomedicine as a distinct therapeutic approach offers new possibilities for treating diseases through mechanical modulation. However, significant challenges remain in translating mechanobiological insights into clinical applications. Success in this endeavor requires continued technological innovation, interdisciplinary collaboration, and careful consideration of therapeutic specificity and safety. As our understanding of cellular mechanics deepens and new tools emerge, mechanomedicine holds the promise of transforming disease treatment through targeted mechanical intervention. The future of medicine may well depend on our ability to harness and manipulate the fundamental physical principles that govern cellular life.

## Data Availability

Data sharing is not applicable to this article as no new data were created or analyzed in this study.
